# Acceptability of tDCS in treating stress-related mental health disorders: a mixed methods study among military patients and caregivers

**DOI:** 10.1186/s12888-021-03086-5

**Published:** 2021-02-15

**Authors:** Fenne M. Smits, Guido J. de Kort, Elbert Geuze

**Affiliations:** 1 Brain Research & Innovation Centre, Ministry of Defence, Lundlaan 1, 3584 EZ Utrecht, The Netherlands; 2 Department of Psychiatry, UMC Utrecht Brain Center, University Medical Center Utrecht, Heidelberglaan 100, 3584 CX Utrecht, The Netherlands

**Keywords:** Noninvasive brain stimulation, tDCS, Acceptability, Treatment coherence, Travel burden, PTSD, Anxiety, Aggression, Caregiver, Military

## Abstract

**Background:**

Noninvasive brain stimulation techniques like transcranial direct current stimulation (tDCS) offer potential new approaches to treat stress-related mental health disorders. While the acceptability of tDCS as a treatment tool plays a crucial role in its development and implementation, little is known about tDCS acceptability for users in mental healthcare, especially in the context of stress-related disorders.

**Methods:**

Using a mixed-methods approach, we investigated tDCS acceptability among 102 active duty and post-active military patients with stress-related symptoms (posttraumatic stress disorder, anxiety and impulsive aggression) who participated in a 5-session tDCS intervention. Quantitative dropout and adverse effects data was collected for all patients involved in the sham-controlled tDCS intervention. We additionally explored perspectives on the acceptability of tDCS treatment via a theory-based semi-structured interview. A subgroup of patients as well as their caregivers were interviewed to include the views of both patients and mental healthcare professionals.

**Results:**

Quantitative outcomes showed minimal tDCS-related adverse effects (mild itching or burning sensations on the scalp) and high tDCS treatment adherence (dropout rate: 4% for active tDCS, 0% for sham). The qualitative outcomes showed predominantly positive attitudes towards tDCS interventions for stress-related disorders, but only as complementary to psychotherapy. Remarkably, despite the perception that sufficient explanation was provided, patients and caregivers stressed that tDCS treatment comprehension was limited and should improve. Also, the travel associated with frequent on-site tDCS sessions may produce a significant barrier to care for patients with stress-related disorders and active-duty military personnel.

**Conclusions:**

Acceptability numbers and perspectives from military patients and caregivers suggest that tDCS is an acceptable complementary tool in the treatment of stress-related disorders. Critically, however, if tDCS is to be used beyond scientific studies, adequately educating users on tDCS working mechanisms is vital to further improve its acceptability. Also, the perceived potential barrier to care due to frequent travel may favor home-based tDCS solutions.

**Trial registration:**

The tDCS intervention was part of a sham-controlled trial registered on 05-18-2016 at the Netherlands Trial Register with ID NL5709.

**Supplementary Information:**

The online version contains supplementary material available at 10.1186/s12888-021-03086-5.

## Background

More than one third of patients with stress-related mental health disorders like posttraumatic stress disorder (PTSD) and anxiety do not benefit from current evidence-based treatments [1, 2], military patients in particular [3–5]. Noninvasive brain stimulation with transcranial magnetic (TMS) or direct current stimulation (tDCS) provides potential add-on treatments or may facilitate effects of pharmacological or psychological therapies [6]. Of these techniques, tDCS might be most suitable to apply in outpatient clinical and military contexts; it is a portable technique, has a better safety profile, and is easier in use [7]. Accordingly, interest for tDCS in the fields of PTSD and anxiety is growing (see e.g. [8, 9]). However, while ongoing studies aim to quantify and optimize tDCS effectivity, the acceptability of tDCS has received remarkably little attention. Especially in the area of stress-related disorders, patients can be particularly skeptical towards alternative treatment approaches [10, 11] and may show lower treatment adherence as a result [12]. Also the views of mental healthcare professionals on tDCS treatment play an important role in its overall acceptability [13]. Hence, it is necessary to understand the acceptability of tDCS as a treatment tool from the perspective of this particular patient population and their caregivers.

TDCS is commonly administered by applying a weak current (~ 1.0–2.5 mA) for 10–40 min between two electrodes placed over the scalp, leading to modulation of neural excitability and plasticity in the underlying cortex [14]. Psychiatric tDCS interventions are often aimed at improving disrupted neurobiological processes involved in (working) memory and emotion regulation [15, 16] and usually comprise 5–30 tDCS sessions applied with an interval of one or several days [17].

Other psychiatric populations show relatively high acceptability for tDCS interventions; investigations among patients with depression, substance use disorders or schizophrenia show that tDCS associated adverse events commonly occur only in a minority of tDCS participants (0–40%) and are restricted to relatively mild sensations including itching, tingling or headache [18]. Dropout rates are low (6–12% [19, 20]) – especially when compared to dropout rates for standard stress-related disorder treatments (e.g., exposure-based psychotherapy: 18–50% [21–24]). The main reasons for dropout are the adverse side effects and missing treatment sessions.

Beside such quantitative measures, a minor body of qualitative research into the acceptability of tDCS is available, conducted in the context of tDCS interventions for stroke rehabilitation and HIV-related depression [25–28]. Here, tDCS participants reported to be satisfied overall with undergoing a tDCS intervention. Yet, the patients also reported to feel some hesitancy towards future tDCS use because of the inflexible tDCS treatment schemes, and burning, itching or painful tDCS sensations (which in general bother patients more than healthy tDCS participants [29]). Importantly, these and other user experiences with novel treatment tools like tDCS can heavily impact its treatment success; the patients’ perspectives on the intervention’s acceptability drive how much they will engage in and adhere to the intervention, and the caregivers’ perspectives partly determine if and how the intervention will be delivered [13, 30]. Early recognition of barriers associated with novel healthcare interventions such as tDCS therefore allows early optimization and cost-effective implementation of the intervention [31].

**Fig. 1:**
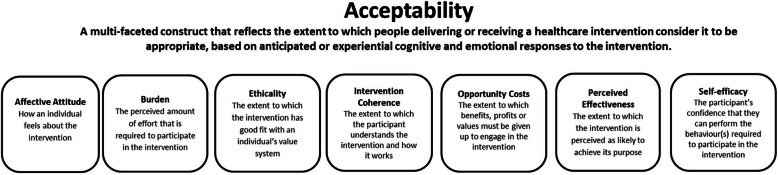
The Theoretical Framework of Acceptability (TFA) [30]. Reproduced under the Creative Commons Attribution 4.0 International License (http://creativecommons.org/licenses/by/4.0/)

Because the acceptability of an intervention is formed through complex (social) interactions between patient, caregiver and technology, this concept is hard to study with quantitative research methods alone [32]. Instead, qualitative examination allows to comprehensively investigate views on the acceptability of a novel intervention. A validated theoretical ground for qualitative assessment of acceptability is offered by the Theoretical Framework of Acceptability (TFA) drafted by Sekhon and colleagues [33]. The TFA is based on extensive research among patients and caregivers [30]. Acceptability is described as: *“the acceptability of an intervention is determined by the appropriateness of addressing the clinical problem, by how well an intervention is suited to an individual lifestyle and how convenient the intervention is able to treat a medical problem”* (Sekhon, Cartwright, & Francis, 2017 p. 6). Figure 1 displays the seven key components that drive acceptability according to the TFA. Using the TFA in qualitative research can thus provide insights in the different aspects of acceptability and underlying reasons, and can be applied to assess the patient’s and the caregiver’s perspective in a similar way.

Here, we studied the acceptability of tDCS for military patients who underwent a tDCS intervention during a period of regular treatment for stress-related disorders like PTSD, anxiety or impulsive aggression. To provide both comparative quantitative measures as well as comprehensive insights into the acceptability of tDCS as a treatment tool, we used a mixed method approach that draws from the strengths of both quantitative and qualitative methods [34]; next to quantifying acceptability in terms of dropout rates and adverse events, we conducted an exploratory study using semi-structured interviews based on the key drivers of the TFA [33] in a subgroup of the participants. Unlike other tDCS acceptability studies, we also included caregivers in the qualitative study to simultaneously gain understanding of the health care professional’s perspectives on tDCS as a treatment tool.

## Method

### Participants and data acquisition

This study was carried out in parallel to a randomized controlled trial (RCT) investigating the effects of prefrontal tDCS combined with cognitive training on PTSD, anxiety and impulsive aggression symptoms. RCT participants were military servicemen and veterans (22–60 years old) of the Dutch Ministry of Defence who received treatment for PTSD, an anxiety disorder or impulsive aggression. Patients with a predominant major depressive disorder diagnosis, a psychotic disorder diagnosis or a history of neurological complaints were excluded from participation. Patients participated in the tDCS intervention between May 2016 and October 2019. The study adhered to CONSORT guidelines where applicable. More details on the RCT protocol were pre-registered at the Netherlands Trial Register (ID: NL5709).

#### Interview respondents and setting

For the qualitative interview study, RCT participants with recent tDCS experience were recruited. Participants were interviewed in the months after they underwent the tDCS intervention (mean time between the tDCS intervention and the interview: 5 months and 4 weeks; range: 1–10 months). We only approached patients who received active tDCS (i.e., no participants from the sham (placebo) condition). Caregivers were recruited among psychologists and psychiatrists from the Dutch military mental healthcare organization who were informed about the tDCS intervention and had treated at least one patient who participated in the tDCS intervention. Interviews were carried out between April and August 2019 and took place at a time and place of the respondent’s preference, usually at the respondent’s home or workplace. Respondents were offered a 10-euro gift card for participation.

### The tDCS intervention

The tDCS intervention in the RCT comprised five tDCS sessions divided over 2 weeks, at the University Medical Center Utrecht, the Netherlands. Patients were allocated to the active tDCS or sham tDCS condition in a 1:1 ratio, based on a MATLAB-generated simple randomization sequence list with codes to activate the DC-stimulator for active or sham tDCS (blind to experimenters and patients). In each session, prefrontal tDCS was applied for 20 min (active) or 16 s (sham), at 1.25 mA, with two 5 × 7 cm electrodes (anode over the right inferior frontal gyrus (IFG), cathode over the left orbital area). During tDCS, patients performed a stop-signal task [35] on a computer for 30 min. Performing the stop-signal task served to activate the tDCS target region (right IFG) and train the cognitive function of inhibitory control. The aim of this tDCS-cognitive training combination was to reduce symptom levels by improving underlying deficits in (the neural network of) inhibitory control over exaggerated or inappropriate behavioral responses (see e.g., [36, 37]). Importantly, although the application of tDCS was always combined with stop-signal task training, the measures assessed in this study focused on the experiences with tDCS. The total duration of each tDCS session was max. one hour. All patients received the tDCS intervention in parallel to regular treatment.

### Data collection

#### Quantitative data collection

For all patients participating in the RCT, we collected data from three quantitative acceptability indicators: dropout rates, adverse effects and changes in emotional state.

(i) Dropout rate (as an indicator of treatment adherence)
  Dropout was defined as not completing the tDCS intervention after starting the first tDCS session.
(ii) Adverse effects of tDCS (as an indicator of treatment burden)
  After each tDCS session, patients filled in the tDCS adverse effects questionnaire [18] by rating on 4-point Likert scales to which extent they had experienced twelve possible tDCS side effects (from 1: “absent” to 4: “severe”), and to which extent they attributed each experienced side effect to tDCS (from 1: “not at all” to 4: “completely”). Also, perceived current strength and tDCS comfort were rated on a 10 cm VAS line with the anchors 0: “Very weak / uncomfortable” and 10: “Very strong / comfortable”. Adverse events occurring outside the research visits were systematically evaluated at each session by asking a description of the adverse event, the adverse event duration, and its severity (1: “mild”, 2: “moderate”, 3: “severe” or 4: “life-threatening”).
(iii) Changes in emotional state (as an indicator of treatment attitude)
  Directly before and after each tDCS session, six emotional state items from the STAI-6 questionnaire [38] were rated by the patient from 1: “Not at all” to 4: “Very much”.

#### Qualitative data collection

Qualitative data was gathered through semi-structured interviews based on the seven key drivers of the TFA [33]. Interviews were held until data saturation was reached. All interviews were recorded with an Olympus VN-8100 PM recorder and transcribed verbatim. Written field-notes containing contextual information (e.g., events happening during the visit) served as additional data source. Interviews lasted on average 31 min.

We used the framework method [39] to analyze the interview data. This systematic and flexible approach for analyzing qualitative data is an iterative process including the following steps: familiarizing with the data by carefully reading the transcripts, deductive coding of concepts in the transcripts according to pre-defined codes (here: based on the TFA key drivers), and inductive coding of concepts in the transcripts by acknowledging emerging new concepts [40]. Two independent other researchers compared our drafted coding scheme to the transcripts. We adapted the coding scheme where needed. A final coding scheme or ‘analytical framework’ [39] was defined according to which all transcripts were coded (see Additional file 1). The coding process was carried out in the qualitative coding software NVivo. Table 1 illustrates an example of the coding process.

**Table 1:** Example of the coding process

Meaningful quote from interview transcript	Pre-defined code	Sub-code	Description
“… I’m not sure if this is going to help me deal with the problem.” [P4]	Ethicality	Relationship between tDCS and symptoms	Comments on how the intervention method fits to the experienced symptoms.
“I’m not much of a talker, so this suits me.” [P1]		Comparison with other therapies	Comments on how well the intervention fits, compared to other treatments.

## Results

### Quantitative results

**Fig. 2:**
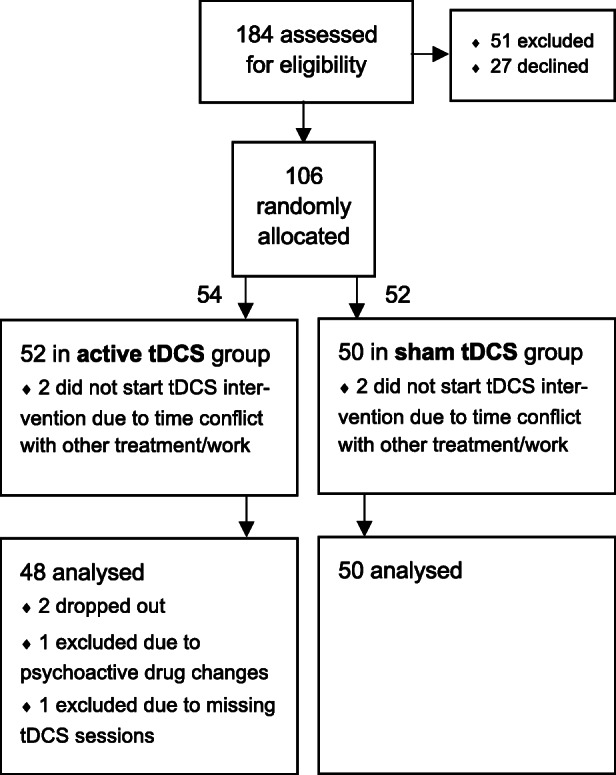
RCT study flow

As depicted in Fig. 2, of the 102 included patients in the tDCS intervention in the RCT, 2 patients in the active tDCS treatment condition dropped out (for reasons, see Table 2). None of the patients in the sham condition dropped out. Hence, treatment adherence was high in both groups.

**Table 2:** Outcomes of the tDCS side effects questionnaire, STAI-6, adverse events and dropout: incidence rates and mean item scores

***Dropout rate***													
	*tDCS*	**4% (*n*** **= 2)**	**Reasons:**	• The first dropout patient got a mild panic attack before the first session, during the tDCS work-up procedure to familiarize participants with tDCS sensations. He preferred to quit the intervention afterwards.									
	*Sham*	**0% (*n*** **= 0)**		• The second dropout patient was admitted to an intensive in-house therapy after the second tDCS session, preventing him from finishing the remaining tDCS sessions of the intervention.									
***Side effects during tDCS sessions (self-report questionnaire)***													
		**Acute change in mood**	**Burning**	**Difficulty concen-trating**	**Dizziness**	**Head ache**	**Itching**	**Nausea**	**Neck pain**	**Pain on the skull**	**Red skin after session**	**Sleepi-ness**	**Tingling**
**Incidence** (severity score > 1)	*tDCS*	18%	46%	67%	16%	29%	55%	4%	19%	11%	3%	55%	54%
	*Sham*	16%	29%	69%	11%	27%	30%	4%	19%	9%	6%	44%	49%
**Attribution to tDCS**	*tDCS*	2,5	3,4	2,1	2,8	2,5	3,3	2,5	1,8	2,9	3,5	2,4	3,1
	*Sham*	2,2	3,0	1,8	2,8	2,1	2,9	3,4	1,5	2,7	3,2	1,9	3,1
***Current strength and comfort during tDCS sessions (self-report questionnaire)***													
		**Perceived current strength**		SD	**Comfort**	SD							
	*tDCS*	**3,5**		2,5	**6,2**	2,2							
	*Sham*	**2,4**		2,2	**6,8**	2,2							
***Adverse events outside the research visits (researcher reports)***													
		**Head ache**	**Nausea**	**Dizziness**	**Fatigue**	**Insomnia**	**Feeling tense**	**Depressed mood**	**Red/sensitive skin at electrode site**				
**Incidence**	*tDCS*	43%	4%	6%	28%	4%	11%	6%	8%				
	*Sham*	41%	8%	12%	14%	8%	20%	6%	4%				
**Severity**		1,5	1,6	1,5	1,9	1,7	1,8	1,8	1,2				
**Duration**		< 1 day	< 1 day	< 1 day	< 1 day	2–3 days	< 1 day	1–2 days	< 1 day				
***Change in emotional state from pre- to post-session (STAI-6)***													
		**Calm**	SD	**Tense**	SD	**Upset**	SD	**Relaxed**	SD	**Content**	SD	**Worried**	SD
	*tDCS*	**-0,1**	0,4	**0**	0,4	**0**	0,2	**0**	0,4	**-0,1**	0,3	**-0,1**	0,2
	*Sham*	**-0,1**	0,3	**+ 0,1**	0,3	**+ 0,1**	0,2	**-0,1**	0,3	**0**	0,3	**-0,1**	0,2

*SD* standard deviation

**Fig. 3:**
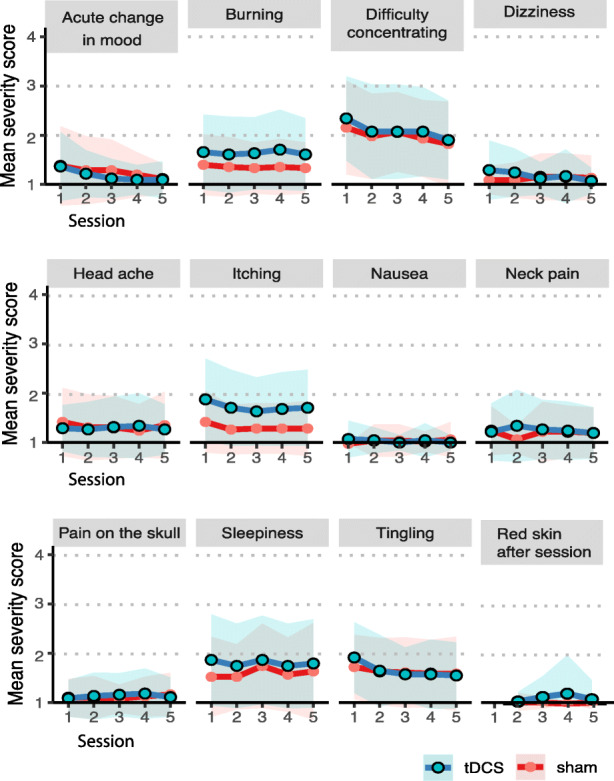
Mean item severity scores on the tDCS adverse effects questionnaire (ribbon: ± standard deviation)

TDCS side effects during the sessions were on average scored as “absent” or “mild”. Of the side effects that were most frequently experienced, the effects that were most strongly attributed to tDCS were: burning, itching and tingling sensations on the scalp (mean attribution score = 3.1). Patients who received active tDCS (vs. sham tDCS) also reported these side effects more frequently (see Table 2) and scored them as slightly more severe (see Fig. 3). Other frequently reported side effects like difficulty concentrating, head ache and sleepiness were also attributed to tDCS, but to a lesser extent (mean attribution score = 2.2) and with a similar incidence across active tDCS and sham groups. All adverse events happening outside of the tDCS sessions that were possibly related to the intervention and reported by more than one participant are listed in Table 2. Adverse events had on average a mild to moderate severity. Head ache after the session was the most prevalent adverse event in both the active tDCS and sham groups (mean incidence: 42%). Patients also experienced fatigue (more frequently in the active tDCS group) and an emotionally or physically “tense” feeling (more frequently in the sham group) after the session. Short periods of dizziness (max. 30 min after the session) were also reported by a minority of patients (9% of all patients, reported more frequently in the sham group). Together these numbers indicate a relatively low burden of adverse events in the tDCS intervention. Compared to the placebo treatment (sham tDCS) the additional adverse effects associated with the real treatment (active tDCS) were very limited.

The average changes in self-reported emotional state (STAI-6) during the tDCS sessions were negligible, see Table 2. This may indicate a neutral attitude of the patients toward the tDCS treatment; the tDCS sessions did not depress or elevate their mood.

The raw data underlying these numbers is provided in Additional file 2.

### Interview respondents

After interviewing 7 patients (3 post-active veterans, 4 active-duty military personnel, age: 26–58 years) and 5 caregivers (age: 27–57 years) data collection was discontinued; the last interviews yielded no new themes among patients or caregivers. For an overview of respondent characteristics, see Table 3.

**Table 3:** Demographic and clinical respondent characteristics

PATIENTS				CAREGIVERS		
Respondent number	Sex	Diagnosis	Current treatment	Respondent number	Sex	Profession
**P1**	male	PTSD	Pharmacological treatment	**CG1**	female	Psychologist
**P2**	male	Aggression regulation problems	Pharmacological treatment, CBT	**CG2**	male	Psychologist
**P3**	male	Anxiety	CBT	**CG3**	female	Psychologist
**P4**	male	Anxiety, Depression	CBT	**CG4**	male	Psychologist
**P5**	male	Anxiety, PTSD, Depression	Pharmacological treatment, CBT	**CG5**	male	Psychiatrist
**P6**	male	PTSD, Aggression regulation problems	EMDR			
**P7**	male	Aggression regulation problems	CBT			

*CBT* cognitive behavioral therapy, *EMDR* eye-movement desensitization and re-processing therapy

### Interview results

The interview results are presented below per key driver of the TFA.

i) Affective Attitude

Most patients and caregivers felt generally positive about the tDCS intervention. To patients, it appealed that the tDCS intervention offered something extra in addition to their regular therapy; they were motivated to do as much as possible to recover from their symptoms.

[“*My motivation was mainly: There is no pain in trying. If it is placebo, it does no harm, and if it is not the placebo then it might, well, give me positive effects.”*] **P5**

Some patients additionally expressed a specific interest in the technological or brain-focused working mechanisms of tDCS, or just wanted to help developing new treatment options. Most caregivers expressed a similar interest, especially towards the cognitive and neurobiological targets of the treatment. Moreover, caregivers recognized that, beside their interest, one of the main reasons for their patients to participate was their desperation to take on ‘any’ treatment available. As a patient stated:

[“*I mean I was very much in need of help. I was a bit desperate, and I thought, you know, I do whatever it takes.”*] **P7**

Some respondents expressed a negative attitude to specific aspects of the intervention. Two patients thought the treatment setting had a ‘low budget’ appearance, mainly due to the look of tDCS equipment (e.g., simple rubber band straps around the head), and to the relatively small, non-modern test room. On the other hand, two other patients specifically mentioned to be content with the treatment setting and the quiet test room. Also, some patients felt unsafe about the treatment before starting the tDCS intervention. A patient expressed this feeling as:

[“*It’s the idea, you know…you’re getting shocks in your head. They are playing with your head.”]* **P3**

Some caregivers pointed out that patients suffering from stress-related disorders are more prone to feelings of unsafety and suspicion, and pointed out that such feelings may pose a barrier to adhering to a tDCS treatment.

ii) Burden

Respondents initially deemed the overall burden of the tDCS intervention low. On a physical level, patients indicated that tDCS associated sensations were tolerated well. Only two patients mentioned a mild burden of headaches or burning and itching sensations after the sessions. On a psychological level, the tDCS sessions were experienced as relatively easy, although the cognitive task during the tDCS sessions was experienced as monotonous and long. Furthermore, the novel and unfamiliar nature of the intervention made a patient feel ill at ease:

[*“When you sit down there, you feel more tense. Then you get the, uhm, current. And you do feel that, yes you do. (…) At a certain moment I felt at ease. But the first few times I didn’t. Then you feel a bit... See, it is all new.”*] **P1**

On a practical level, both patients and caregivers pointed out that a 5-session tDCS intervention is a low burden, especially when compared to regular treatment schemes. However, for some patients traveling towards the hospital posed a high burden, as traveling caused them a lot of anxiety and stress. Also patients with a short travel time declared that a longer travel time would cause a higher burden. Caregivers indeed pointed out that for patients with stress-related disorders travel time should be as short as possible. One patient explained:

[“*I got very aggressive in traffic. (…) And the train is even worse. There, people don’t do what you want. So, transport from A to B in a crowded space is quite a problem.”*] **P6**

He later added:

[*“But you can’t send it* [the tDCS equipment] *home as a package and say: Here you are, do this.”*] **P6**

Furthermore, one patient suggested to offer tDCS participants some time to ‘recover’ after each tDCS session, to relieve the potential tension caused by the session and to feel more secure to travel home.

iii) Opportunity costs

The opportunity costs of the tDCS intervention were deemed low by all respondents. Because all patients were allowed time off from work for treatment, patients didn’t perceive that the time invested in participation posed opportunity costs at the moment, but most of them anticipated higher costs with heavier intervention schemes or full-time job obligations. In addition, some caregivers noted the potential difficulty in treatment adherence in this specific population due to military training and operations abroad.

iv) Ethicality

Patients particularly appreciated that the tDCS intervention did not trigger negative thoughts or fearful memories, in contrary to the exposure in psychotherapy. For some patients, the tDCS sessions even offered distraction from negative thoughts or anxious feelings. Some patients therefore ascribed a high ethicality value to the tDCS intervention, and would favor tDCS over psychotherapy if a tDCS treatment would be equally effective. As one patient noted:

[“*You don’t have to put everything on the table, you don’t have to dig stuff up. It is fast and comfortable.”*] **P6**

However, at the same time, both patients and caregivers expressed the expectation that tDCS would only ‘work’ in combination with psychotherapy. All respondents deemed it necessary for recovery to talk about their mental health problems and the underlying causes. One patient also mentioned he missed social therapeutic interaction during the intervention.

A second theme that emerged from the caregivers’ perspective is the particular suitability of tDCS treatments for military personnel because of its ‘high-tech’ feel, which may lower the barrier to treatment:

[“*The association with cyber, space, earplugs. I think that* [the technology] *is a benefit for this subpopulation.”*] **CG2**

xxii) Perceived effectiveness

Most patients perceived no significant effect of tDCS on their symptoms. Because all patients received psychological or pharmacological treatment in parallel, patients who reported improvements in mental health after the tDCS intervention could not specifically attribute this to tDCS.

Caregivers acknowledged a potential of the technology as add-on to existing treatments for this patient group. However, caregivers in general did not expect tDCS effects to be ‘ground-breaking’, especially not when stress-related symptoms are caused by more complex underlying issues, e.g., related to childhood trauma or personality.

Some patients did report short-term improvements in their ability to focus, cognitive ‘clarity’, or a generally calmer mood. These short-term effects disappeared directly after the tDCS session or in the days afterwards.

vi) Coherence

Most patients and caregivers felt they were adequately informed about the tDCS intervention:

[*“It was all explained to me quite well. And you do a test beforehand* [an impulse control task]*, and then you know what to expect. In practice, it’s more or less the same.”*] **P2**

[*“And* [the researcher] *took a lot of effort to explain it.”*] **CG1**

In sharp contrast, however, all patients and most caregivers expressed a lack of sufficient understanding of the tDCS intervention. The same caregiver (CG1), for example, continued to say:

[*“And then you think: I remember so little of it. I just find it a bit shocking how little I know about it.”*] **CG1**

A patient mentioned:

[“*I don’t know how it works. The only thing I know is that they gave me a screen, and I had to push buttons.”*] **P3**

(Pushing buttons refers to the cognitive task during the tDCS sessions.)

The majority of respondents reported a general feeling of incomprehension towards the clinical mechanism of action; the relationship between the tDCS intervention and the disorder-specific symptoms was unclear to most respondents. As one patient put it:

[*“Then* [after the first tDCS session] *I thought: Well, I’m not sure if this is going to help me deal with the problem.”*] **P4**

A number of caregivers and patients pointed out that the neurobiological working mechanisms of tDCS were hard to grasp for them.

In response to this treatment incomprehension, a second theme emerged, comprising the importance of treatment coherence. According to the patients, better comprehension of how tDCS works and how tDCS can affect their symptoms is critical because it would (i) reduce feelings of stress resulting from not knowing what effects to expect, (ii) improve their personal contribution to facilitate the treatment’s effects, and (iii) increase motivation to adhere to the treatment. One caregiver elucidated why treatment coherence is especially important for patients with stress-related disorders:

[*Very important. It can be a vehicle for participating in a state-of-the-art treatment. Because then, they can trust it. And only then they can ‘surrender’ to treatment.’*] **CG3**

Another caregiver conceived it critical that caregivers should fully understand the treatment’s mechanism of action, also because the patient’s decision to participate in a novel healthcare intervention often depends on the opinion of the caregiver.

Two patients suggested to explain the working mechanisms of tDCS in a simpler manner and making use of ‘imagery’.

vii) Self-efficacy

Patients overall felt capable to adhere to all of the intervention components. Caregivers did also not foresee capability problems associated with the tDCS intervention.

Yet, although not directly related to tDCS, two patients reported the inability to maintain focus during the cognitive task, and two patients encountered difficulty in comprehending the written information and questionnaire items.

## Discussion

The acceptability of novel treatments such as tDCS contributes significantly to its successful implementation in clinical practice. If tDCS is to play an important role in treating stress-related disorders, its acceptability in this context is important to understand. This mixed methods study is the first to examine the acceptability of tDCS as a treatment tool for stress-related mental health disorders from both the patient and caregiver perspective. We gathered quantitative measures of acceptability in an RCT with 102 military patients undergoing a 5-session tDCS intervention, including the dropout rate, adverse side effects and emotional responses to the tDCS sessions. In an additional exploratory study, we carried out semi-structured interviews based on the TFA [33] to gather in-depth information on the full range of views and experiences with tDCS among a subgroup of the patients and a group of caregivers.

In summary, the quantitative outcomes showed relatively high acceptability of tDCS; treatment adherence was high and only mild adverse sensations on the scalp could be directly attributed to tDCS, conform recently updated tDCS adverse effects profiles [41]. This was supported by the qualitative outcomes, showing that the affective attitude towards the tDCS intervention was predominantly positive. Also, the burden and opportunity costs were deemed low and self-efficacy was high. Regarding ethicality, tDCS fitted well into the value system of the respondents, although the technique was mainly perceived as complementary to psychotherapy. Strikingly, however, the tDCS intervention coherence was very limited among patients as well as caregivers. Furthermore, the applied short tDCS intervention was not perceived as effective to treat the stress-related symptoms. A higher travel barrier was anticipated for more intensive treatment schemes. Below, we further discuss the major findings.

The most notable finding was the mismatch in perspectives on treatment coherence. Although patients and caregivers expressed their impression that sufficient explanation of the study and intervention had been provided, almost all respondents showed limited comprehension of the clinical mechanisms of action of tDCS. This may be related to unfamiliarity with the neurobiological processes targeted by tDCS. Patients and caregivers both emphasized the importance of understanding the working mechanisms of a tDCS intervention and its intended impact on clinically relevant outcomes. Respondents anticipated that better understanding could improve the affective attitude towards the technique, lower the barrier to participate and increase treatment adherence. Low treatment coherence also seemed to negatively impact the ethicality, as some patients expressed that they didn’t know how the tDCS intervention would ‘help them’. A negative impact of low treatment coherence on other acceptability aspects is consistent with previous findings. For example, limited understanding of psychotherapy processes also induces skepticism towards the treatment among patients and caregivers [42, 43]. User’s expectations may also directly influence tDCS effectiveness [44]. Appropriately educating users on tDCS thus appears vital for its acceptability and effectivity as a treatment tool. This likely also applies to brain stimulation tools like TMS and other novel (neurobiological) treatment options.

Respondents found no significant burden or opportunity costs in a tDCS intervention. Contrary to previous findings [25], our patients perceived only mild adverse side effects of tDCS and did not experience tDCS sensations as a burden or barrier. Interestingly, besides the itching or burning sensations on the scalp, most adverse effects could not directly be attributed to active tDCS, suggesting that such adverse effects (e.g., head ache) are linked to general RCT participation rather than to active tDCS itself. However, a potential burden was perceived in travelling towards the hospital for the tDCS sessions; travelling can be a severe trouble for patients with stress-related disorders, and specifically for this population also during military training or operational activities abroad. Veterans with PTSD in general seem to regard frequent visits as a disadvantage of treatment [45]. To lower the travel barrier, taking more advantage of the technique’s portability and further developing home-based tDCS is highly recommended. Despite some obstacles (e.g., adverse effects due to misuse [46–48]), the feasibility of home-based tDCS is already increasing [49, 50]. Also, home-based tDCS may have additional potential to treatment in the post COVID-19 era.

Regarding the ethicality of the tDCS intervention, patients and caregivers were positive for different reasons. The caregivers expected that the intervention’s ‘technological feel’ could appeal to military patients and thereby lower treatment barriers. Indeed, incorporating technological methods that appeal to a population may be beneficial for psychiatric treatment [51]. Instead, the patients particularly appreciated the low emotional burden of the tDCS intervention, i.e., the possibility of treatment without exposure to trauma or feared situations. Correspondingly, less focus on verbal communication and lower perceived stress during treatment sessions are also particularly appreciated aspects in EMDR therapy for stress-related disorders [43, 45], while trauma exposure is experienced as a struggle in regular psychotherapy [42, 52]. For military PTSD patients, exposure to trauma during therapy even poses a major barrier to psychotherapeutic treatment [53]. On the other hand, patients were also uncertain about how well the tDCS intervention could address their symptoms. Neither patients nor caregivers believed that a technique like tDCS can completely replace psychotherapy or ‘talking’. In fact, ‘talking’, personal contact and the role of the caregiver are regarded as the most important aspects of psychotherapy [43] that positively contribute to willingness to participate in research (especially among traumatized patients [54]), treatment acceptability [52, 55], therapeutic effectivity, and self-efficacy in managing symptoms [42, 52, 55–58]. Accordingly, treatments for PTSD and anxiety without these interpersonal aspects (such as medication) are commonly prescribed only as add-on to psychotherapy [59]. Taken together, the technical feel and minimal emotional burden of a tDCS intervention might be useful to lower the barrier to seek treatment, but our findings suggest that tDCS for stress-related disorders should ultimately be offered in addition to an interpersonal treatment option like psychotherapy.

Furthermore, the respondents’ positive attitude towards participating in the intervention stemmed mainly from a general motivation to explore alternative treatment options. Hope for recovery and desire for treatment innovation were also the main reasons for tDCS participation in a previous study [28]. Notably, the characteristics of the equipment and treatment setting had a significant influence on patients’ affective attitude, either in a positive or negative way. The technique also induced some initial feelings of unsafety among patients. As also pointed out by the caregivers, patients with stress-related disorder are prone to anxious feelings prior to starting a novel treatment [42, 52, 55, 57] and may prefer treatments they are familiar with [45]. To improve the affective attitude towards tDCS as a professional treatment tool, attention should be paid to the appearance and comfort of the equipment and treatment setting, and to patients’ understanding of the treatment and its safety profile.

Finally, patients expressed the feeling that many more tDCS sessions would be needed in order to effectuate a clinically significant change. The perceived clinical effects of this short tDCS intervention on symptoms were very limited or completely absent. Yet, in line with previously reported experiences with tDCS [26, 28], patients perceived increased focus and cognitive ‘clarity’ during tDCS or in the hours afterwards.

### Study strengths and limitations

This study extended knowledge on tDCS acceptability to the context of stress-related disorders, military patients and, importantly, to the level of the caregiver. Furthermore, the relevance and reliability of our findings were maximized by combining quantitative data with qualitative outcomes in a mixed methods approach, and by using a validated theoretical framework and analysis method for the qualitative data. We therefore believe that these results make an important contribution to insights in the acceptability of tDCS in mental health care.

Yet, our study met a number of limitations. First, we investigated a sample of military and mainly male respondents. All respondents were also individuals who voluntarily participated in an RCT. Our results may represent the specific views of this population, although we believe that the most important findings are generalizable to other patients with stress-related disorders (e.g., regarding the difficulty comprehending tDCS working mechanisms and the travel burden). Second, no new themes emerged in the last interviews, indicating that the most important themes are covered by the data. However, the sample size of the interview study was relatively low, especially compared to the sample size of the quantitative study. Future studies are needed to confirm our qualitative findings in larger samples and other populations. Third, it should be mentioned that our findings are closely connected with the characteristics of the tDCS intervention as applied in our study. For example, the cognitive training on the stop-signal task should be seen as part of the total experience of the tDCS intervention. This may have influenced the experience with tDCS itself. Likewise, some of our findings may be very study-specific, such as aspects of the affective attitude (e.g., regarding tDCS equipment) and perceived effectiveness, which can limit the generalizability of our findings towards other types of tDCS interventions. It should also be noted that the quantitative measures were taken during the tDCS intervention, while the interviews were conducted one or more months after tDCS participation. Although respondents did generally not report to have large issues with remembering specific details of the tDCS intervention, the qualitative data depended on the accuracy of the participants’ memory recall. In this respect, there is a discrepancy between the quantitative and qualitative data; the numbers reflect immediate tDCS experiences, while the interview results reflect overarching retrospective perspectives on tDCS as a technique for stress-related disorder treatment.

## Conclusion

In this study we investigated the acceptability of tDCS for the first time in the context of stress-related disorder treatment. High treatment adherence and minimal adverse side effects reflected high acceptability of tDCS. Exploratory findings on the subjective perspectives of military patients and their professional caregivers also showed that tDCS is overall regarded as an acceptable complementary treatment tool for stress-related disorders. However, our respondents raised two major issues. First, limited understanding of how tDCS works as a treatment tool highlighted the need to improve treatment comprehension. The essence of treatment comprehension was further emphasized by its negative influence on the affective attitude and perceived suitability of tDCS to treat stress-related symptoms. Second, travelling for treatment visits potentially poses an important barrier in this population. This barrier will grow when more (frequent) sessions are required for clinical effectiveness. Although the results reported here are closely connected with the way tDCS was applied in our study, they highlight that efforts should be made to better educate tDCS users and further develop home-based tDCS solutions to secure optimal tDCS acceptability and, in turn, intervention success.

## Supplementary Information

**Additional file 1.**

**Additional file 2.**

## Data Availability

All quantitative data analyzed during this study are included in this published article and its supplementary information files.

## Abbreviations

CBT: Cognitive behavioral therapy
EMDR: Eye movement desensitization and reprocessing
PTSD: Posttraumatic stress disorder
RCT: Randomized-controlled trial
STAI-6: Six-item short-form of the State-Trait Anxiety Inventory
tDCS: transcranial direct current stimulation
TFA: Theoretical framework of acceptability
TMS: Transcranial magnetic stimulation
