# Supplementary figures and images for: Acceptability of tDCS in treating stress-related mental health disorders: a mixed methods study among military patients and caregivers
Source: BMC Psychiatry. 2021 Feb 15;21:97. doi: 10.1186/s12888-021-03086-5 (PMC7883955; doi:10.1186/s12888-021-03086-5)

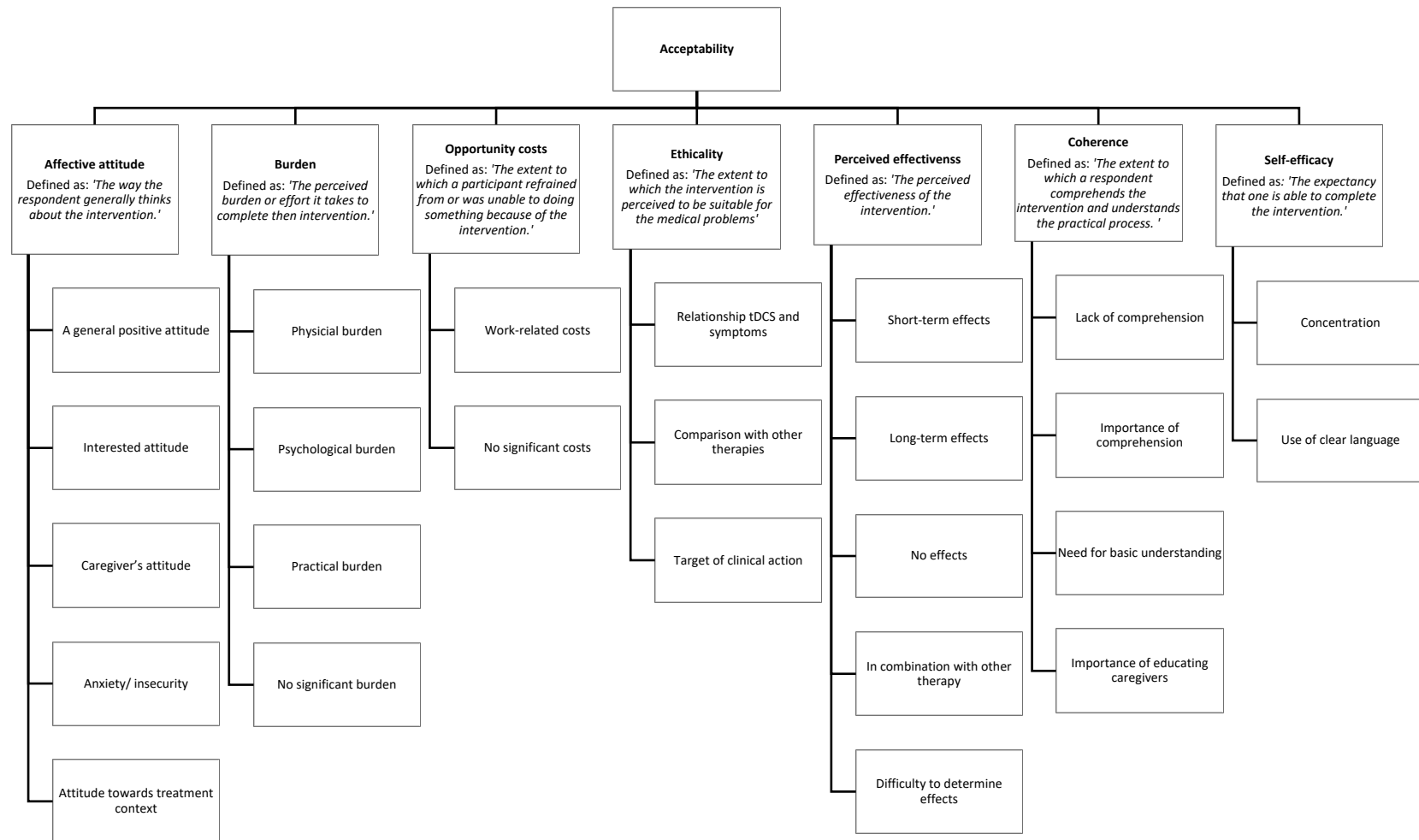

Final coding scheme.

Supplement: Supplementary file 1 — Additional file 1. [file 12888_2021_3086_MOESM1_ESM.pdf]
